# Hemoptysis—Error of Diagnosis

**Published:** 1887-08

**Authors:** 


### Hemoptysis—Error of Diagnosis.

At the meeting of the New York Surgical Society, May 11, 1887, Dr. Charles McBurney mentioned the case of a gentleman who had suffered for twelve years with "hemoptysis," and had been all over Europe, frequently having his chest examined, under the impression that he had phthisis. No evidence of pulmonary trouble could be discovered, and, as none of his physicians had ever chanced to see him when he was bleeding, the cause of the hemorrhage could not be discovered. Dr. McBurney happened to examine him while he was spitting blood, and found that it came from the bottom of a tooth-cavity. He was cured by removal of the tooth.

THE alteration of vulcanized rubber, according to Pharmacist Major Balland, is owing to the formation of sulphuric acid, resulting from the slow oxidation of the sulphur employed for vulcanizing. To prevent the destructive change, he recommends a simple washing of the objects with plain water or water slightly alkaline, repeated every two or three months.

